# Systematic review of mediterranean diet interventions in menopausal women

**DOI:** 10.3934/publichealth.2024005

**Published:** 2024-01-10

**Authors:** Carla Gonçalves, Helena Moreira, Ricardo Santos

**Affiliations:** 1 CITAB - Centre for the Research and Technology of Agro-Environmental and Biological Sciences, Institute for Innovation, Capacity Building and Sustainability of Agri-food Production (Inov4Agro), Universidade de Trás-os-Montes e Alto Douro, Vila Real, Portugal; 2 EPIUnit - Instituto de Saúde Pública - Laboratório para a Investigação Integrativa e Translacional em Saúde Populacional (ITR), Universidade do Porto, Porto, Portugal; 3 CISAS - Center for Research and Development in Agrifood Systems and Sustainability, Instituto Politécnico de Viana do Castelo, 4900-347 Viana do Castelo, Portugal; 4 CIDESD - Research Center in Sports Sciences, Health Sciences and Human Development, Laboratory of Biomechanics, Body Composition and Health (LaB2Health), Universidade de Trás-os-Montes e Alto Douro, Vila Real, Portugal; 5 CIFI2D - Centre of Research, Education, Innovation and Intervention in Sport, Faculdade de Desporto, Universidade do Porto, Porto, Portugal

**Keywords:** mediterranean diet, menopause, women's health, interventional studies

## Abstract

The increasing lifespan of women and their extended time spent in menopause pose significant challenges for health care systems, primarily due to the impacts of postmenopausal estrogen deficiency and aging on health. Menopause's onset is linked to a heightened prevalence of obesity, metabolic syndrome, cardiovascular disease, and osteoporosis. Diet is particularly relevant during menopause given its impact on quality of life and longevity and its modifiability. Because the Mediterranean diet is currently regarded as one of the healthiest dietary models in the world, the aim of this systematic review was to assess current evidence regarding the effectiveness of studies on the Mediterranean diet as an intervention for menopausal women. A systematic review of intervention-based studies involving the Mediterranean diet among menopausal women was performed in Scopus, PubMed, and Web of Science. The results of seven that met the inclusion criteria suggests that adherence to the Mediterranean diet can have beneficial impacts on menopausal women's health, including reductions in weight, blood pressure, blood ω6: ω3 ratio, triglycerides, total cholesterol, and LDL levels. Those results seem to be relevant for public health interventions aimed at improving menopausal women's quality of life.

## 1. Introduction

Menopause is a physiological process marked by the date of a woman's last menstruation that indicates definitive ovarian failure. Menopause is diagnosed retrospectively after 12 consecutive months of amenorrhea without any other pathological or physiological cause [1]. The age at which menopause occurs, generally between 45 and 55 years old, is primarily determined by genetics but also susceptible to the influence of environmental factors such as obesity, multiparity, physical activity, tobacco use, and alcoholism [2].

For women, entering into menopause can be associated with an increased prevalence of obesity, metabolic syndrome, cardiovascular disease, and osteoporosis [3]. Against those risks, diet is particularly relevant given its impact on quality of life and longevity and its modifiability [4]. Because menopause is also viewed as a time in women's lives when they are more susceptible to changing habits and acquiring healthier behaviors [1], it presents an excellent opportunity for health interventions as well.

Of all components of lifestyle, nutrition exerts some of the most significant impacts on postmenopausal women's quality of life and morbidity. Higher adherence to the Mediterranean diet (MD) has been shown to improve the prevention and management of age-associated non-communicable diseases, including cardiovascular and metabolic diseases, neurodegenerative diseases, cancer, depression, respiratory diseases, and fragility fractures [5]. In turn, the MD may also reduce total mortality in the population [6] and lengthen lifespans [7]. Therefore, the MD, currently recognized as one of the healthiest dietary models worldwide [8], may offer many benefits to women in the climacteric phase of life. The MD is characterized by a relatively high intake of vegetable products (i.e., vegetables, fruit, low-refined cereals, legumes, nuts, olives, and olive oil); the moderate consumption of fish, white meat, eggs, and dairy products (preferably in the form of cheese and yogurt); and the relatively low consumption of red and processed meat and wine with meals [9]. Given those characteristics, individuals with better adherence to the dietary pattern also have a better nutritional intake profile, including a high intake of fiber, vitamins, minerals, and phytochemicals (e.g., flavonoids, polyphenols, and carotenoids), along with a low glycemic index, a high monounsaturated: saturated fat intake ratio, and low omega-6: omega-3 fatty acid intake ratio [10].

Considering all of the above, the aim of this systematic review was to examine current evidence about the effectiveness of studies on MD-based interventions conducted among menopausal women.

## 2. Materials and methods

### 2.1. Search strategy

A systematic search of peer-reviewed studies published up to July 2023 was conducted following the PRISMA guidelines [11], however, the study protocol had not been previously published. The search used the following databases: Scopus, PubMed, and Web of Science. The query question was (“intervention” or “food and nutrition education” or “trial” or “pilot study” or “program”) AND (“mediterranean diet”) AND (“menopause” or “menopausal”). Only studies published in the last 15 years were included. Reference lists of eligible studies were scanned to identify additional pertinent publications, and the bibliographic reference management software EndNote™ 20 software (Clarivate, Philadelphia, USA) was used.

### 2.2. Inclusion and exclusion criteria

All studies that provided the description of interventions with MD in menopausal women published in the last 15 years (2008–2023) were included. This encompassed randomized trials, pilot intervention without a control arm or experimental study. Exclusion criteria included studies not exclusively targeted on dietary intervention but involving various health behaviors (e.g., physical activity), as well as protocols, commentaries, and opinion papers. Articles were not excluded because of age, sample size, sampling methodology, or geographical location. The titles and abstracts of retrieved articles were independently evaluated by two researchers (CG and RS), not blinded to authorships.

### 2.3. Data extraction and analysis

Relevant information was extracted regarding the country, participants (number, age, and inclusion and exclusion criteria), study design, enrolment (start and end date), length of participant intervention and follow-up, as well as recruitment and sampling procedures, intervention description, and outcomes. When extracting data related to outcomes, the authors opted to focus on the parameters most mentioned in the literature regarding anthropometric/body composition parameters (weight, body mass index (BMI), total fat mass, visceral fat, fat-free mass, and waist circumference), blood pressure (systolic blood pressure (SBP) and diastolic blood pressure (DBP)), dietary (MD adherence, energy, fat, saturated fat, polyunsaturated fatty acids (PUFA), monosaturated fatty acids (MUFA), ω6: ω3 ratio, carbohydrates and fiber), and blood outcomes (ω6: ω3 ratio, triglycerides, total cholesterol, high density lipoproteins (HDL), low density lipoproteins (LDL), fasting glucose, homocysteine, C-reactive protein (CRP), and nitric oxide level (NO)). Data extraction was carried out by one researcher, CG, in consultation with a second researcher, RS, when required.

### 2.4. Studies quality assessment

The quality of randomized intervention studies were assessed by the revised Cochrane risk-of-bias tool for randomized trials (RoB 2 tool) [12]. This tool evaluates bias in five domains: (1) randomization process, (2) deviations from intended interventions, (3) missing outcome data, (4) measurement of the outcome, and (5) selection of the reported results. The possible risk-of-bias judgements in each domain are low risk of bias, some concerns, or high risk of bias. Furthermore, an overall risk-of-bias judgement of the study was done giving the evaluation of each domain. Studies were considered to have a high risk of bias if they were judged to be at least in one domain with high risk of bias. They were considered to have some concerns of bias if they were judged to raise some concerns for at least one domain. They were considered low risk of bias only if they were judged to be at low risk of bias for all domains.

The quality of non-randomised intervention studies were assessed by “Risk Of Bias In Non-randomised Studies - of Interventions” (ROBINS-I tool) [13]. This tool considered the assessment of bias in seven domains: (1) confounding, (2) selection of participants, (3) classification of interventions, (4) deviations from intended interventions, (5) missing data, (6) measurement of outcomes, and (7) selection of the reported result. The categories for risk of bias judgements are low, moderate, serious, and critical risk of bias. An overall risk-of-bias judgement was determined to be low risk of bias if the study was low risk for all domains, moderate risk of bias if the study was of low or moderate risk for all domains, serious risk of bias if the study was at serious risk in at least one domain, and critical risk of bias if the study was at critical risk in at least one domain. The risk-of-bias plots were generated using the app robvis [14].

## 3. Results

**Figure 1.**
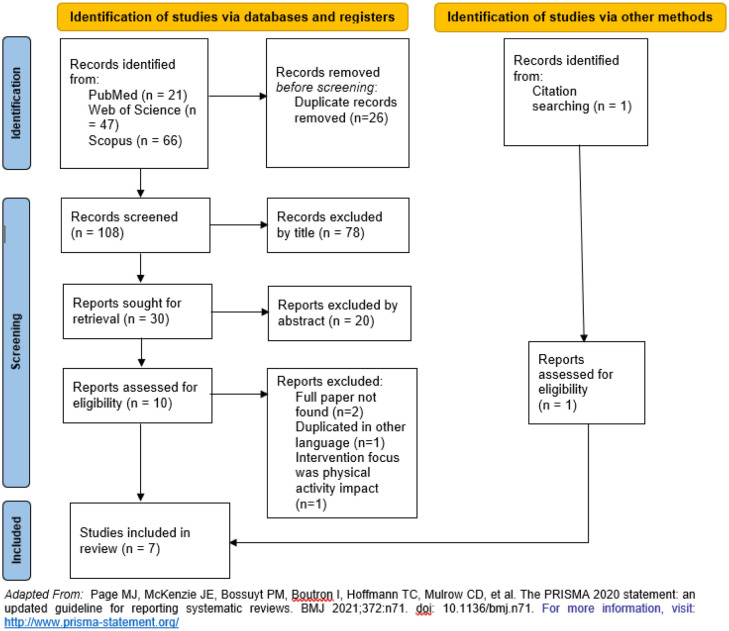
Flow diagram of the process to identify and screen included studies (PRISMA flow diagram).

The first search yielded 134 potentially relevant publications (Figure 1). Following the removal of duplicates and title and abstract screening, 10 publications were retrieved for full-text screening, and at the end six were included in the systematic review. One study was included resulting from the reference list of eligible studies.

From the seven intervention studies, three were conducted in countries bordering the Mediterranean (Spain [15] and Italy [16],[17]), three were from of Northern Europe (Poland [18]–[20]) and one was from USA [21]. The number of participants varies between 16 and 230, and all studies included menopausal women with mean ages ranging from 49–77 years old (Table 1). The supplementary table provide details in inclusion and exclusion criteria for participants selection.

Two studies were non-randomized intervention studies. One was a controlled before-and-after study involving 16 menopausal women following the MD [21] and the other was a case-control study, comparing menopausal women (case) with fertile women over 45 years old (control) intervein with MD [16]. Five studies were randomized trials, randomizing participants to the MD or usual diet [15], or placebo [17], or Central European diet (CED) [18]–[20] (Table 2). The follow-up ranged from 2 months to 1 year, and the intervention spanned from 1 week to 1 year. The intervention designs are diverse, and included education workshops [15], prescription of the MD by a nutritionist [16],[21], oral supplementation with enriched extra virgin olive oil [17], and the provision of pre-packaged main meals according to MD plus dietary advice [18]–[20].

**Table 1.** Characteristics of participants from included studies (*n* = 7).

**First Author, publication year**	**Study design**	**Country**	**Participants**		**Arms**	**Age (years, *mean* ± *sd*)**	**BMI (kg/m^2^, *mean* ± *sd*)**
			**Randomized**	**Analysed**			
**Bihuniak JD, 2016 [21]**	Controlled before-and-after study	United States of America	-	16	One arm: MD	77.0±6.8	26.1±3.1
**Rodríguez AS, 2016 [15]**	Randomized clinical trial	Spain	320	230	Arm 1: intervention group MD Arm 2: control group	53.2±4.3	27.6±5.5 27.3±5.3
**Vignini A, 2017 [17]**	Randomized clinical trial	Italy	60	60	Arm 1: intervention group supplemented with oral extra virgin olive oil (VOO) enriched with vitamins D3, K1 and B6. Arm 2: control group with oral supplementation with VOO	54.6±3.7 55.6±2.6	25.2±2.7 25.9±3.1
**Bajerska J, 2018 [20]**	Randomized clinical trial	Poland	144	130	Arm 1: Mediterranean diet (MED) Arm 2: Central European diet (CED)	60.3±4.7 60.8±4.7	33.8±5.6 33.6±4.2
**Duś-Żuchowska M, 2018 [18]**							
**Muzsik A, 2019 [19]**							
**Lombardo M, 2020 [16]**	Case-control study	Italy	89	89	Arm 1: Menopausal women Arm 2: Fertile women over 45 years of age	57.1±4.9 48.8±4.0	30.6±5.4 29.5±5.0

Note: BMI: Body mass index; DRI: Dietary reference intake; CVD: Cardiovascular diseases; NR: Not reported; MD: Mediterranean diet; CED: Central European diet; VOO: Virgin olive oil.

**Table 2.** Intervention description and types of outcomes from included studies (*n* = 7).

**First author, publication year**	**Intervention length**	**Follow-up (months)**	**Recruitment**	**Randomization**	**Intervention characteristics**	**Outcomes**				
						**Anthropo-metric**	**Dietary**	**Blood**	**Blood pressure**	**Genetic**
**Bihuniak JD, 2016 [21]**	12 weeks	2 years	Convenience	No	Advised by a dietitian on how to follow a MD, and substitute sources of saturated fat and refined carbohydrates by extra virgin olive oil (3 T/day), walnuts (1.5 oz/day), and fatty fish (3–5 servings/wk) which were provided at 3-week intervals.	Yes	Yes	Yes	No	No
**Rodríguez AS, 2016 [15]**	1 week	1 year	Recruited in two primary health centres in one province	Yes	*Intervention group*: receive 3 interactive educational workshops (cardiovascular disease, maintenance of healthy habits and MD). *Control group*: participants received information pamphlets about MD.	Yes	Yes	No	No	No
**Vignini A, 2017 [17]**	1 year	1 year	Recruitment was performed in the ambulatory centre of the local hospital	Yes	*Intervention group*: oral supplementation with 20 mL/d extra virgin olive oil (VOO) enriched with vitamins D3 (100% RDA), K1 (100% RDA), and B6 (30% RDA). *Control group*: oral supplementation with 20 mL/d VOO. Standardized MD prescribed for both groups.	Yes	No	Yes	No	No
**Bajerska J, 2018 [20]**	16 weeks	16 weeks	Recruited through advertisements	Yes, stratified for BMI	Both groups were advised to follow a hypocaloric diet without added salt, refined fats and sugar; and to maintain the usual level of physical activity. During the intervention period, participants picked up packaged main meals and the other meals were prepared by the participants according to the prescribed instructions. *MED group*: followed a MD food plan. Olive oil and nuts were consumed every day. The proportion of soluble to insoluble fiber was 20–80%. *CED group*: followed a CED food plan, with a special focus on high levels of fiber. The proportion of soluble to insoluble fiber was 35–65%.	Yes	Yes	Yes	Yes	No
**Duś-Żuchowska M, 2018 [18]**						No	No	Yes	No	No
**Muzsik A, 2019 [19]**						No	No	Yes	No	Yes
**Lombardo M, 2020 [16]**	2 months	2 months	Convenience, recruited in nutrition clinic	No	All participants were advised to follow a two-month hypocaloric, moderate fat and MD balanced diet individually tailored, with the use of herbs and spices instead of salt, reduction of red meat to less than twice a week and to eat fish at least twice a week. The main sources of daily added fat were 25–35 g of olive oil and 20 g of nuts per day. It was recommended to only performed minimal aerobic training.	Yes	No	Yes	Yes	No

Note: NR: Not reported; MD: Mediterranean diet; BMI: Body mass index; CED: Central European diet group; MED: Mediterranean diet group; VOO: Virgin olive oil

The included studies aimed to assess the effect of the MD on various parameters, including the reduction of weight [15],[16],[20] and changes in body composition [15], cardiovascular risk factors [17],[19]–[21], atherosclerosis prevention [18], and bone health [17]. To assess these effects, studies evaluated several outcomes that authors summarize in Table 3 (anthropometric and blood pressure outcomes), Table 4 (dietary outcomes), and Table 5 (blood outcomes). One study also evaluates the association between genetic polymorphism of FADS1 and FADS2 genes and fatty acids concentrations after MD intervention period [19].

In general, MD was modestly efficacious at reducing body weight (ranging from -0.2 to -7.7 kg)[16],[20],[21], with significant changes only in one study [20]. No significant effects on BMI were found. Regarding body composition, one study reported a significant reduction in total fat mass (mean change -6.6 kg) [20], discrepant changes were found in visceral fat between studies (one with significant increase [15] and another with significant decrease [20]). A reduction on fat free mass (ranging from -0.6 to -1.1 kg) [16],[20] was noted, with only in one study achieving significant changes [20]. Waist circumference (ranging from -0.1 to -7.4 cm), SBP (ranging from -9 to -10.2 mmHg) and DBP (ranging from -6.7 to -7 mmHg) decreases after MD intervention [15],[16],[20], however significant changes only found in one study [20] (Table 3).

Three studies evaluated dietary outcomes (Table 4) [15],[20],[21]. One of these studies present MD adherence score from MEDAS-14 questionnaire [22], while the others reported the MD adherence score from MedSD questionnaire [23] and nutritional analysis from multiple 3-day diet records [21] or from daily food records [20]. In both studies [15],[21] MD interventions significantly increased the adherence to the MD, increased the intake of fat, PUFA, MUFA, and decreased the intake of saturated fat, ω6: ω3 ratio and carbohydrates. However, there was discrepancies in the findings related to energy intake. Bihuniak et al. [21] reported that energy intake increased and Bajerska et al. [20] found a significant decrease.

Changes in lipid levels were verified in five studies [16],[17],[19]–[21] (Table 5). It was verified that MD led to a decrease in triglycerides (ranging from -33.9 to -5.8 mg/dL), total cholesterol (ranging from -22.8 to -2.3 mg/dL), LDL (ranging from -28.2 to -4.0 mg/dL), and ω6: ω3 ratio (ranging from -1.5 to -0.2). The effect of MD on HDL, yielded different results, with two studies reporting an increase in HDL values [16],[17] and the other two showing a decrease in HDL values [20],[21]. Bajerska et al. [20] verified a significant increase in homocysteine levels (mean change +0.7 µM, *p* = 0.002), this could be relevant since some authors correlates homocysteine with cardiovascular problems [24]. MD also showed significant and beneficial impact on C-reactive protein (CRP) (mean change -1.2 mg/L) [18] and on nitric oxide level (NO) (mean change -6.6 nmol NO/mg protein) [17], both indicators of CVD risk, due to the development of atherosclerosis that is associated with inflammation within the vessel walls.

The MD produced similar effects on glycemic control (Table 5), since all of them showed reduction on fasting glucose levels (ranging from -6.4 to -0.8 mg/dL) [16],[17],[20]. Notably, the study from Bajerska et al. showed significant decrease on fasting glucose levels and in homeostatic model assessment of insulin resistance (HOMA2-IR) in women submitted to MD (mean change -0.46, *p* < 0.0001) [20].

**Table 3.** Changes in body composition and blood pressure outcomes presented in included studies.

**First Author, publication year**	**Body Composition**												**Blood Pressure**			
	**Weight (kg)**		**Body Mass Index (Kg/m^2^)**		**Total fat mass (kg)**		**Visceral fat (kg)**		**Fat-free mass (kg)**		**Waist circumference (cm)**		**SBP (mmHg)**		**DBP (mmHg)**	
	**Baseline**	**Mean change**	**Baseline**	**Mean change**	**Baseline**	**Mean change**	**Baseline**	**Mean change**	**Baseline**	**Mean change**	**Baseline**	**Mean change**	**Baseline**	**Mean change**	**Baseline**	**Mean change**
**Bihuniak JD, 2016 [21]**	65.5±9.5	-0.2#	26.1±3.1	-0.5#	NR	NR	NR	NR	NR	NR	NR	NR	NR	NR	NR	NR
**Rodríguez AS, 2016 [15]**	IG NR	NR	27.6±5.5	+0.1	24.8±9.9	+0.2	7.8±3.0	+0.2*	NR	NR	92.9±13.3	-0.1	NR	NR	NR	NR
	CG NR		27.3±5.3	+0.5*	24.8±9.2	+1.3*	7.7±2.8	+0.5*			93.4±13.6	+1.6*				
**Vignini A, 2017 [17]**	PlaVOO NR	NR	25.2±2.7	-0.2#	NR	NR	NR	NR	NR	NR	NR	NR	NR	NR	NR	NR
	VitVOO NR		25.9±3.1	-1.7#												
**Bajerska J, 2018 [20]**	MED 87.0±NR	−7.7*	NR	NR	40.4±NR	−6.6*	1.1±NR	−0.3*	46.6±NR	−1.1*	105.0±NR	−7.4*	140.9±NR	−10.2*	87.0±NR	−6.7*
	CED 85.3±NR	−7.5			39.5±NR	−6.7	1.1±NR	−0.3	45.8±NR	−0.8*	105.4±NR	−7.5	142.1±NR	−10.4	86.9±NR	−8.1
**Lombardo M, 2020 [16]**	MEN 80.8±16.2	-3.7	30.6±5.4	-0.9	33.4±11.1	-2.4	NR	NR	45.1±5.4	-0.6	97.5±15.3	-3.1	133.8±17.2	-9	85.2±13.0	-7
	REG 77.0±14.1	-3.1	29.5±5.0	-0.8	29.9±9.7	-2.2			44.9±5.3	-0.8	92.2±11.5	-3.2	124.6±13.3	-6.8	80.6±12.8	-3.3

Note: Values are reported as: mean ± standard deviation, unless otherwise specified. NR: Not reported; NO: Values are not an outcome; TEI: Total energy intake; PUFA: Polyunsaturated fatty acids; MUFA: Monounsaturated fatty acids; VitVO: Vitaminized virgin olive oil group; PlaVOO: Placebo virgin olive oil group; MED: Group with mediterranean diet; CED: Group with central european diet; IG: Intervention group; CG: Control group; MEN: Menopausal women group; REG: Not menopausal; SBP: Systolic blood pressure; DBP: Diastolic blood pressure; #: mean change was computed as the difference in means between baseline and follow-up using data from the publication; *p < 0.05.

**Table 4.** Changes in dietary outcomes presented in included studies.

**First Author, publication year**	**Dietary**																	
	**Med Diet adherence**		**Energy (kcal/d)**		**Fat (%TEI)**		**Saturated fat (%TEI)**		**PUFA (%TEI)**		**MUFA (%TEI)**		**ω6: ω3 ratio**		**Carbohydrates (%TEI)**		**Fiber (g)**	
	**Baseline**	**Mean change**	**Baseline**	**Mean change**	**Baseline**	**Mean change**	**Baseline**	**Mean change**	**Baseline**	**Mean change**	**Baseline**	**Mean change**	**Baseline**	**Mean change**	**Baseline**	**Mean change**	**Baseline**	**Mean change**
**Bihuniak JD, 2016 [21]**	32.3±4.3	+9.0#*	1679±231	+124#	31.4±5.6	+9.3#*	10.3±2.4	-0.7#	NR	NR	NR	NR	8.2±5.2	-3.7#*	51.5±7.2	-7.0#*	20.3±7.4	+1#
**Rodríguez AS, 2016 [15]**	IG 7.06±2.02	+2.31#*	NR	NR	NR	NR	NR	NR	NR	NR	NR	NR	NR	NR	NR	NR	NR	NR
	CG 6.96±2.15	+0.15																
**Bajerska J, 2018 [20]**	MED NR	NR	1912±NR	-574*	36.1±NR	+0.3*	16.9±NR	-8.4*	5.2±NR	+3.8*	14.0±NR	+5.0	NR	NR	47.5±NR	-2.5*	21.0±NR	+11.7*
	CED NR	NR	1936±NR	-574	35.9±NR	-8.5	17.4±NR	-8.6	5.2±NR	+3.9	13.2±NR	-3.9			48.6±NR	+5.7	21.7±NR	+17.6

Note: Values are reported as: mean ± standard deviation, unless otherwise specified. NR: not reported; TEI: Total energy intake; PUFA: Polyunsaturated fatty acids; MUFA: Monounsaturated fatty acids; MED: Group with mediterranean diet; CED: Group with central european diet; IG: Intervention group; CG: Control group; #: mean change was computed as the difference in means between baseline and follow-up using data from the publication; *p < 0.05.

**Table 5.** Changes in blood outcomes presented in included studies.

**First author, publication year**	**Blood**																	
	**ω6: ω3 ratio**		**Triglycerides (mg/dL)**		**Total cholesterol (mg/dL)**		**HDL (mg/dL)**		**LDL (mg/dL)**		**Fasting glucose (mg/dL)**		**Homocysteine (µM)**		**CRP (mg/L)**		**NO (nmol NO/ mg protein)**	
	**Baseline**	**Mean change**	**Baseline**	**Mean change**	**Baseline**	**Mean change**	**Baseline**	**Mean change**	**Baseline**	**Mean change**	**Baseline**	**Mean change**	**Baseline**	**Mean change**	**Baseline**	**Mean change**	**Baseline**	**Mean change**
**Bihuniak JD, 2016 [21]**	10.4±1.9	-1.5#*	99.8±34.8	-12.5 #*	205.5±31.9	-2.3#	68.1±13.0	-4.0#*	117.6±25.6	-4.0#	NR	NR	NR	NR	NR	NR	NR	NR
**Vignini A, 2017 [17]**	NR	NR	VitVO 125.2±46.9	-5.8#	219.6±34.3	-15.3#	58.6±14.1	+7.1#*	133.1±33.7	-5.5#	82.5±9.0	-0.8#	NR	NR	NR	NR	43.8±4.9	-6.6#*
			PlaVO 127.2±44.3	-12.0#	218.3±33.5	-12.7#	57.6±15.3	+6.8#*	132.4±32.4	-3.9#	83.5±9.0	-2.0#					41.9±4.2	+0.7#
**Bajerska J, 2018 [20]**	MED NR	NR	157.4±NR	-33.9*	235.8±NR	-15.5*	55.3±NR	-0.1	149.7±NR	-9.4	98.0±NR	-6.4*	11.5±NR	+0.7*	NR	NR	NR	NR
	CED NR	NR	164.1±NR	-38.8	226.4±NR	-11.2	53.1±NR	-2.0	143.1±NR	-4.9	98.1±NR	-5.4	10.9±NR	+0.8	NR	NR	NR	NR
**Duś-Żuchowska M, 2018 [18]**	NR	NR	NR	NR	NR	NR	NR	NR	NR	NR	NR	NR	NR	NR	MED 4.5±4.5	-1.2*	NR	NR
															CED 4.5±4.4	-0.9*		
**Muzsik A, 2019 [19]**	MED 2.5±0.7∥	-0.2	NR	NR	NR	NR	NR	NR	NR	NR	NR	NR	NR	NR	NR	NR	NR	NR
	CED 2.4±0.7∥	+0.01																
**Lombardo M, 2020 [16]**	NR	NR	MEN 108.3±65.2	-22.9	219.6±41.7	-22.8	58.3±12.6	+6.0	143.6±36.2	-28.2*	92.7±14.0	-2.5	NR	NR	NR	NR	NR	NR
			REG 92.0±39.0	+8.2	202.9±35.5	-9.3	58.9±16.4	+2.2	130.3±29.4	-7.7	92.4±9.4	-2.2						

Note: Values are reported as: mean ± standard deviation, unless otherwise specified. NR: Not reported; HDL: High density lipoprotein; LDL: Low density lipoprotein; CRP: C-reactive protein; NO: Nitric oxide level; MED: Mediterranean diet group; CED: Central european diet group; MEN: Menopausal women group; REG: Not menopausal; VitVO: Vitaminized virgin olive oil group; PlaVO: Placebo virgin olive oil group; IG: Intervention group; CG: Control group; ∥: values obtained in red blood cells; #: mean change was computed as the difference in means between baseline and follow-up using data from the publication; *p < 0.05.

**Figure 2.**
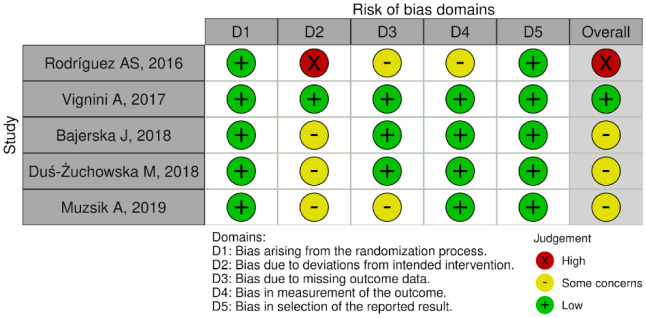
Risk of bias in randomized intervention studies.

The five randomized studies included exhibited degrees of bias of low concern [17], some concerns [18]–[20], and one with high concern [15] (Figure 2). The domain with poorest performance in the analysis was the bias related to deviations from intended interventions.

**Figure 3.**
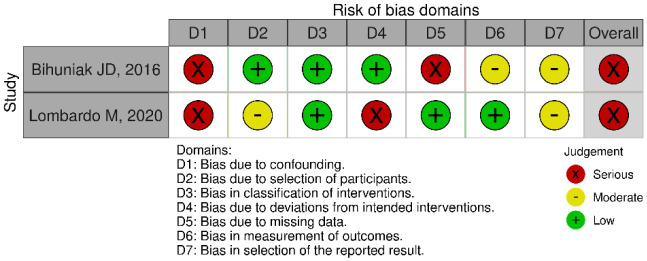
Risk of bias in non-randomized intervention studies.

The two non-randomized studies showed a serious risk of bias [16],[21], mainly due to confounding variables (e.g., physical activity level), deviations from intended interventions, and missing data (Figure 3).

## 4. Discussion

In 2020, the United Nations estimated that, worldwide, 985 million women were 50 years old or older, and that number is projected to increase to 1.65 billion by 2050 [25]. Therefore, women are living longer, and the phase of their life lived in menopause is increasing as well. In that context, the immediate and long-term impacts of estrogen depletion and aging on health pose a significant challenge to health care systems around the world.

Optimizing diet is increasingly recognized as being crucial in strategies aimed to promote women's health during the menopause period [4]. The MD, characterized by the high intake of plan-based foods, is considered by several authors to be a possible explanation for improved bone metabolism, enhanced muscle performance, and reduced oxidative stress, inflammation, and insulin resistance [4],[26]. Therefore, gaining insights into the impacts associated with adhering to the MD on menopausal women's health is essential for the appropriate and specific design of public health interventions.

This systematic review, addressing the effectiveness of MD-based interventions conducted among menopausal women, suggests that adhering to the MD can have beneficial impacts during the climacteric phase of life, including the reduction of weight, blood pressure, blood ω6: ω3 ratio, triglycerides, total cholesterol, and LDL levels.

Menopause has been associated with an increase in body weight and changes in body composition such as an increase in fat mass and a reduction in fat-free mass. That shift is attributed to the reduction of basal metabolic rate, diminished physical activity, and the loss of estrogen's influences on lipoprotein lipase activity and lipolysis [27],[28]. Moreover, changes in body composition reflect the significant decline in muscle mass, with a sharp annual decline after the age of 50 years, ranging between 0.6% and 2%, most prominently in the first 3 years after the onset of permanent amenorrhea [29],[30]. Those changes can be critical for the risk of insulin resistance, diabetes, and cardiovascular diseases. The studies included in this systematic review suggest that following the MD is associated with reduced body weight but cannot reduce BMI and can exert conflicting impacts on body composition (e.g., total and visceral fat levels). The observed discrepancies in results regarding BMI may be partially explained by the reduction in women's height, often linked to worsened bone condition in the spine, such that osteoporotic vertebral fractures are common in the postmenopause period but are seldom clinically identified [31]. Other authors have found that the MD can result in greater weight loss than a low-fat diet [32] and that a higher adherence to the diet is associated with increased likelihood of maintained weight loss [33] and reduced abdominal adiposity, particularly visceral fat levels [34],[35]. However, the particularities of menopausal women can be complex, and total energy intake and physical activity level can be important confounders that should be more rigorously controlled in future studies.

The accelerated loss of fat-free mass, also reported to occur during menopause, increases the risk of sarcopenia, a condition that can lead to functional impairment and physical disabilities [36]. The accelerated loss of fat-free mass reflects a decline in both muscle mass and bone mass, the latter of which intensifies after menopause [37]. The results of this review generally show the reduction of fat-free mass in menopausal women following a MD-based dietary intervention. Moreover, although no clinical trials were found that examined adherence to the MD and sarcopenia, a systematic review of observational studies has shown no evidence of the MD's positive effect on sarcopenia other than its general positive role in improving muscle mass and muscle function [38].

During the reproductive period, the production of oestrogens exerts a protective effect on endothelial function and lipid metabolism, with a decrease in LDL and the ratio of total cholesterol and HDL. Estrogen, along with changes in the lipid profile, contributes to improved vasodilation and reduced levels of homocysteine and fibrinogen [39]. Conversely, the drop in estrogen levels in the bloodstream may potentially contribute to an increase in blood pressure through various mechanisms, such as influencing the arterial wall, activating the renin–angiotensin system, and stimulating the sympathetic nervous system [40]. Those physiological changes are associated with and elevated risk of cardiovascular diseases and the higher prevalence of metabolic syndrome [4]. The results of this review suggest that menopausal women who follow the MD may experience benefits such as reduced blood pressure, triglycerides, total cholesterol, and LDL levels. Those results align with the findings of other authors [32],[41],[42] and can be partly explained by components of the MD such as a high prevalence of unsaturated fats in sources of fiber and protein, a scarcity of saturated fats, and a richness of fruits, vegetables, whole grains, nuts, and legumes, all of which contribute to the dietary pattern [41]. Other authors have also found that supplementation of fish oil rich in omega-3 significantly improves endothelial function and reduces pro-inflammatory markers among patients with diabetes due to its antioxidant and anti-inflammatory properties [43].

Increased blood pressure with aging can be also explained by increased sensitivity to salt with aging, which contributes to the development of age-related cardiovascular disease [44]. The MD, however, makes no prescriptions regarding salt intake, and Viroli et al. [45] did not detect any differences in sodium intake between a lower versus higher adherence to the MD. Another recent systematic review concluded that among individuals more than 65 years old, high potassium intake (i.e., >3510 mg/d) and a low sodium: potassium ratio (i.e., <1) have a beneficial effect on reducing the risk of cardiovascular disease. That positive outcome is largely because the potassium present in fruits and vegetables can mitigate the harmful effects of excess sodium intake [46].

A systematic review encompassing all meta-analyses and randomised controlled trials that compared the MD with a control diet on the treatment of type 2 diabetes and prediabetic states found that MD was associated with better glycemic control and improved cardiovascular risk factors than control diets, including those characterized by lower fat content [47]. That association was attributed to components of the MD that are considered to be anti-inflammatory and antioxidative (e.g., fiber, vitamins, and minerals, as well as antioxidants and polyphenols), coupled with a lower intake of proinflammatory foods and nutrients (e.g., saturated and trans fatty acids, refined sugars, and starches). Those explanations align with the findings of the present review, which all showed a reduction in fasting glucose levels among menopausal women [16],[17],[20].

It may be surprising that the present systematic review focused on the effectiveness of dietary interventions but only three studies assessed dietary outcomes [15],[20],[21], primarily to control adherence to the intervention. That point is significant because most health-related outcomes can be directly influenced by differences in the daily total energy and nutritional intake of participants. Two of the studies evaluated adherence to the MD using two different scoring systems [15],[21], which could also be viewed as a limitation. After all, using a score to estimate a dietary pattern is limited by subjectivity, which can cause considerable variability in the interpretation of the results [48].

Another limitation of the systematic review was that most studies did not include assessments of physical activity or total energy intake, both of which could be significant confounders affecting the outcomes. Thus, the interpretation of the results should be approached with caution. Beyond that, the relatively short duration of interventions (e.g., 1 week) could be insufficient to achieve meaningful results.

## 5. Conclusions

In conclusion, this systematic review suggests that adhering to a MD can have beneficial impacts on menopausal women's health, including the reduction of weight, blood pressure, blood ω6: ω3 ratio, triglycerides, total cholesterol, and LDL levels. Those findings appear to be relevant in the context of public health interventions to aimed to enhancing the quality of life for menopausal women.

## Use of AI tools declaration

The authors declare they have not used Artificial Intelligence (AI) tools in the creation of this article.



## References

[1] Cavadas LF, Nunes A, Pinheiro M (2010). Management of menopause in primary health care. Acta Med Port.

[2] Ceylan B, Özerdoğan N (2015). Factors affecting age of onset of menopause and determination of quality of life in menopause. Turk J Obstet Gynecol.

[3] Nappi RE, Simoncini T (2021). Menopause transition: A golden age to prevent cardiovascular disease. Lancet Diabetes Endocrinol.

[4] Silva TR, Oppermann K, Reis FM (2021). Nutrition in menopausal women: A narrative review. Nutrients.

[5] Di Daniele N, Noce A, Vidiri MF (2017). Impact of Mediterranean diet on metabolic syndrome, cancer and longevity. Oncotarget.

[6] Trichopoulou A, Costacou T, Bamia C (2003). Adherence to a Mediterranean diet and survival in a Greek population. N Engl J Med.

[7] Pes GM, Dore MP, Tsofliou F (2022). Diet and longevity in the Blue Zones: A set-and-forget issue?. Maturitas.

[8] Morris L, Bhatnagar D (2016). The Mediterranean diet. Curr Opin Lipidol.

[9] Barbosa C, Real H, Pimenta P (2017). Roda da alimentação mediterrânica e pirâmide da dieta mediterrânica: comparação entre os dois guias alimentares. Acta Portuguesa de Nutrição.

[10] Castro-Quezada I, Román-Viñas B, Serra-Majem L (2014). The mediterranean diet and nutritional adequacy: A review. Nutrients.

[11] Page MJ, Moher D, Bossuyt PM (2021). PRISMA 2020 explanation and elaboration: Updated guidance and exemplars for reporting systematic reviews. BMJ.

[12] Higgins JP, Savović J, Page MJ (2019). Assessing risk of bias in a randomized trial. Cochrane handbook for systematic reviews of interventions.

[13] Sterne JA, Hernán MA, Reeves BC (2016). ROBINS-I: A tool for assessing risk of bias in non-randomised studies of interventions. BMJ.

[14] McGuinness LA, Higgins JPT (2021). Risk-of-bias VISualization (robvis): An R package and Shiny web app for visualizing risk-of-bias assessments. Res Synth Methods.

[15] Rodriguez AS, Soidan JLG, Santos MDT (2016). Benefits of an educational intervention on diet and anthropometric profile of women with one cardiovascular risk factor. Medicina Clinica.

[16] Lombardo M, Perrone MA, Guseva E (2020). Losing weight after menopause with minimal aerobic training and mediterranean diet. Nutrients.

[17] Vignini A, Nanetti L, Raffaelli F (2017). Effect of 1-y oral supplementation with vitaminized olive oil on platelets from healthy postmenopausal women. Nutrition.

[18] Duś-Zuchowska M, Bajerska J, Krzyzanowska P (2018). The central European diet as an alternative to the mediterranean diet in atherosclerosis prevention in postmenopausal obese women with a high risk of metabolic syndrome-A randomized nutritional trial. Acta Sci Pol Technol Aliment.

[19] Muzsik A, Bajerska J, Jeleń HH (2019). FADS1 and FADS2 polymorphism are associated with changes in fatty acid concentrations after calorie-restricted Central European and Mediterranean diets. Menopause.

[20] Bajerska J, Chmurzynska A, Muzsik A (2018). Weight loss and metabolic health effects from energy-restricted mediterranean and Central-European diets in postmenopausal women: A randomized controlled trial. Sci Rep.

[21] Bihuniak JD, Ramos A, Huedo-Medina T (2016). Adherence to a mediterranean-style diet and its influence on cardiovascular risk factors in postmenopausal women. J Acad Nutr Diet.

[22] Martínez-González MA, García-Arellano A, Toledo E (2012). A 14-item mediterranean diet assessment tool and obesity indexes among high-risk subjects: The PREDIMED trial. PLoS One.

[23] Panagiotakos DB, Pitsavos C, Stefanadis C (2006). Dietary patterns: A mediterranean diet score and its relation to clinical and biological markers of cardiovascular disease risk. Nutr Metab Cardiovasc Dis.

[24] Ganguly P, Alam SF (2015). Role of homocysteine in the development of cardiovascular disease. Nutr J.

[25] Olén NB, Lehsten V (2022). High-resolution global population projections dataset developed with CMIP6 RCP and SSP scenarios for year 2010–2100. Data Brief.

[26] Silva TRd, Martins CC, Ferreira LL (2019). Mediterranean diet is associated with bone mineral density and muscle mass in postmenopausal women. Climacteric.

[27] Al-Safi ZA, Polotsky AJ (2015). Obesity and menopause. Best Pract Res Clin Obstet Gynaecol.

[28] Greendale GA, Sternfeld B, Huang M (2019). Changes in body composition and weight during the menopause transition. JCI Insight.

[29] Maltais ML, Desroches J, Dionne IJ (2009). Changes in muscle mass and strength after menopause. J Musculoskelet Neuronal Interact.

[30] Aloia JF, McGowan DM, Vaswani AN (1991). Relationship of menopause to skeletal and muscle mass. Am J Clin Nutr.

[31] Forsmo S, Hvam HM, Rea ML (2007). Height loss, forearm bone density and bone loss in menopausal women: a 15-year prospective study. The Nord-Trøndelag Health Study, Norway. Osteoporos Int.

[32] Mancini JG, Filion KB, Atallah R (2016). Systematic review of the Mediterranean diet for long-term weight loss. Am J Med.

[33] Kim JY (2021). Optimal diet strategies for weight loss and weight loss maintenance. J Obes Metab Syndr.

[34] Muscogiuri G, Verde L, Sulu C (2022). Mediterranean diet and obesity-related disorders: what is the evidence?. Curr Obes Rep.

[35] Dinu M, Pagliai G, Casini A (2018). Mediterranean diet and multiple health outcomes: An umbrella review of meta-analyses of observational studies and randomised trials. Eur J Clin Nutr.

[36] Buckinx F, Aubertin-Leheudre M (2022). Sarcopenia in menopausal women: Current perspectives. Int J Womens Health.

[37] Finkelstein JS, Brockwell SE, Mehta V (2008). Bone mineral density changes during the menopause transition in a multiethnic cohort of women. J Clin Endocrinol Metab.

[38] Papadopoulou SK, Detopoulou P, Voulgaridou G (2023). Mediterranean diet and sarcopenia features in apparently healthy adults over 65 years: A systematic review. Nutrients.

[39] Antunes S, Marcelino O, Aguiar T (2003). Fisiopatologia da menopausa. Revista Portuguesa de Medicina Geral e Familiar.

[40] Taddei S (2009). Blood pressure through aging and menopause. Climacteric.

[41] Widmer RJ, Flammer AJ, Lerman LO (2015). The Mediterranean diet, its components, and cardiovascular disease. Am J Med.

[42] Tuttolomondo A, Simonetta I, Daidone M (2019). Metabolic and vascular effect of the mediterranean diet. Int J Mol Sci.

[43] Rizza S, Tesauro M, Cardillo C (2009). Fish oil supplementation improves endothelial function in normoglycemic offspring of patients with type 2 diabetes. Atherosclerosis.

[44] Paar M, Pavenstädt H, Kusche-Vihrog K (2014). Endothelial sodium channels trigger endothelial salt sensitivity with aging. Hypertension.

[45] Viroli G, Gonçalves C, Pinho O (2021). High adherence to Mediterranean diet is not associated with an improved sodium and potassium intake. Nutrients.

[46] Goncalves C, Abreu S (2020). Sodium and potassium intake and cardiovascular disease in older people: A systematic review. Nutrients.

[47] Esposito K, Maiorino MI, Bellastella G (2015). A journey into a Mediterranean diet and type 2 diabetes: A systematic review with meta-analyses. BMJ Open.

[48] Sofi F, Cesari F, Abbate R (2008). Adherence to Mediterranean diet and health status: Meta-analysis. BMJ.

