# Supplementary material for: Systematic review of mediterranean diet interventions in menopausal women
Source: AIMS Public Health. 2024 Jan 10;11(1):110–29. doi: 10.3934/publichealth.2024005 (PMC11007410; doi:10.3934/publichealth.2024005)
Supplement: Supplementary file 1 [file publichealth-11-01-005-s001.pdf]

Public Health

AIMS Public Health, 11(1): 110–129.
DOI: 10.3934/publichealth.2024005
Received: 20 September 2023
Revised: 21 December 2023
Accepted: 25 December 2023
Published: 10 January 2024

http://www.aimspress.com/journal/aimsph

Review

Systematic review of mediterranean diet interventions in menopausal women

Carla Gonçalves[1,2,3,*], Helena Moreira[1,4] and Ricardo Santos[5]

[1] CITAB - Centre for the Research and Technology of Agro-Environmental and Biological Sciences, Institute for Innovation, Capacity Building and Sustainability of Agri-food Production (Inov4Agro), Universidade de Trás-os-Montes e Alto Douro, Vila Real, Portugal
[2] EPIUnit - Instituto de Saúde Pública - Laboratório para a Investigação Integrativa e Translacional em Saúde Populacional (ITR), Universidade do Porto, Porto, Portugal
[3] CISAS - Center for Research and Development in Agrifood Systems and Sustainability, Instituto Politécnico de Viana do Castelo, 4900-347 Viana do Castelo, Portugal
[4] CIDESD - Research Center in Sports Sciences, Health Sciences and Human Development, Laboratory of Biomechanics, Body Composition and Health (LaB2Health), Universidade de Trás-os-Montes e Alto Douro, Vila Real, Portugal
[5] CIFI2D - Centre of Research, Education, Innovation and Intervention in Sport, Faculdade de Desporto, Universidade do Porto, Porto, Portugal

* **Correspondence:** Email: carlagoncalves.pt@gmail.com.

Supplementary

Table S1. Inclusion and exclusion criteria of participants reported in included studies (*n* = 7).

**First Author, publication year**	**Inclusion and exclusion criteria**
Bihuniak JD, 2016 [18]	Exclusion: any disease that may affect bone metabolism, cancers of any kind (except basal or squamous cell of the skin) during the past 5 years, use of medication that affect bone metabolism, physical activity more than 75 min/day for 6 days/wk, dietary behaviours or supplementation in excess of DRI upper limits: total vitamin D excess of 10,000 IU/day or <400 IU/day, total calcium exceeding 2,000 mg/day or <400 mg/day, following a medically prescribed diet or dietary pattern similar to the MD, history of chronic renal or liver disease, history of hip fracture or known vertebral fracture within the past year, alcoholic beverage intake more than 3 drinks/d, allergy to fish or nuts, achieving a score >81% on the Mediterranean Diet Score assessment form, consumption of > 5 servings/d of fruit or vegetables, consumption ≥ 2 servings/wk of fatty fish, or consumption of ≥ 3 servings/wk of seafood.
Rodríguez AS, 2016 [12]	Inclusion: women have at least one cardiovascular risk factor (diabetes mellitus, dyslipidaemia and/or hypertension).<br><br>Exclusion: refuse to participate, have some disease or psychical situation that was considered impeditive.
	Inclusion: postmenopausal women (1 y after last menses); normal screening laboratory values; non-smokers; non-alcoholic; BMI in the range of 22 to 28 kg/m2.<br><br>Exclusion: decompensated diseases of the liver, kidney, pancreas, lung, or heart; a history of active cancer in the past 5 y; chronic disease (e.g., diabetes mellitus, CVD, cancer, or fat malabsorption syndromes), abnormal bleeding, or thrombotic disorders; regularly taking nonsteroidal anti-inflammatory drugs or drugs known to alter platelet function or the homeostatic system in general.
Bajerska J, 2018 [17]	Inclusion: postmenopausal (at least 1 year since the last menstrual period plus a follicle-stimulating hormone concentration of more than 30 IU/L at screening); with central obesity (waist circumference ≥ 80 cm) plus at least one other criterion of metabolic syndrome; non-smoking.
Duś-Żuchowska M, 2018 [15]	
Muzsik A, 2019 [16]	Exclusion: type 2 diabetes; monogenic dyslipidaemia; a history of CVD; use of hypoglycaemic, hypolipidemic, anti-inflammatory, or weight loss agents, as well as any drug known to influence liver function; with endocrine disorders or on hormonal replacement therapy; significant weight change in the 6 months prior to the study, intolerance or food allergy to key components of the intervention diets, alcohol consumption >2 drinks/day.
Lombardo M, 2020 [13]	Inclusion: an age ≥ 45; BMI between 25 and 45 kg/m2; a stable body weight over the 2 months before the baseline; no change in medication for at least 3 months.<br>Exclusion: pregnancy or nursing; diabetes mellitus; chronic kidney disease; glucocorticoids, oestrogens and anti-convulsant therapies; history of CVD, neoplastic or other systemic disease; any medication use known to affect weight/energy expenditure; alcohol intake >3 drinks/d.

AIMS Press

© 2024 the Author(s), licensee AIMS Press. This is an open access article distributed under the terms of the Creative Commons Attribution License (http://creativecommons.org/licenses/by/4.0)
